# Adaptive Evolution of Cooperation through Darwinian Dynamics in Public Goods Games

**DOI:** 10.1371/journal.pone.0025496

**Published:** 2011-10-25

**Authors:** Kuiying Deng, Tianguang Chu

**Affiliations:** 1 State Key Laboratory for Turbulence and Complex Systems, College of Engineering, Peking University, Beijing, China; 2 Department of Mathematics, Uppsala University, Uppsala, Sweden; 3 Key Laboratory of Machine Perception (Ministry of Education), Peking University, Beijing, China; Hungarian Academy of Sciences, Hungary

## Abstract

The linear or threshold Public Goods game (PGG) is extensively accepted as a paradigmatic model to approach the evolution of cooperation in social dilemmas. Here we explore the significant effect of nonlinearity of the structures of public goods on the evolution of cooperation within the well-mixed population by adopting Darwinian dynamics, which simultaneously consider the evolution of populations and strategies on a continuous adaptive landscape, and extend the concept of evolutionarily stable strategy (ESS) as a coalition of strategies that is both convergent-stable and resistant to invasion. Results show (i) that in the linear PGG contributing nothing is an ESS, which contradicts experimental data, (ii) that in the threshold PGG contributing the threshold value is a fragile ESS, which cannot resist the invasion of contributing nothing, and (iii) that there exists a robust ESS of contributing more than half in the sigmoid PGG if the return rate is relatively high. This work reveals the significant effect of the nonlinearity of the structures of public goods on the evolution of cooperation, and suggests that, compared with the linear or threshold PGG, the sigmoid PGG might be a more proper model for the evolution of cooperation within the well-mixed population.

## Introduction

The evolution of cooperation in social dilemmas has attracted broad interests across disciplines [1]–[5]. Social dilemmas are situations in which individual rationality leads to collective irrationality [6], [7]. They are pervasive in all kinds of relationships, from the interpersonal to the international. For example, a public local library financed through donations benefits all people in the community. One can benefit most if he donates nothing. However, if everyone reasoned like this, the library would not keep running due to the lack of finance, and all people would be worst off [8]. This is a Public Goods dilemma. There exists another kind of social dilemma called commons dilemma. For example, farmers living in a common grassland can benefit more by raising as many cattle as they want. However, if every farmer reasoned like this, the grassland would be depleted very soon, and all farmers would worst off [6]. The same reasoning applies to these two kinds of social dilemmas, so we focus on the Public Goods dilemma, which is usually modeled as a *Public Goods game* (PGG).

**Figure 1:**
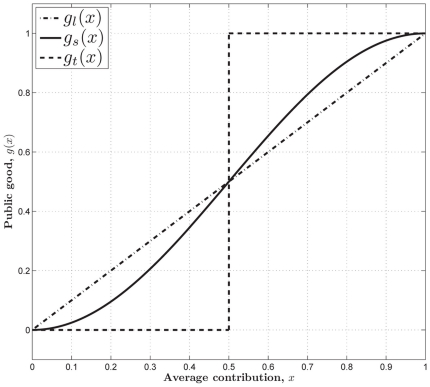
The three kinds of structures of the PGG. (**Dash-dot**) The linear PGG, 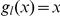. (**Solid**) The sigmoid PGG, 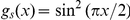. (**Dashed**) The threshold PGG, 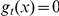 if 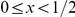, and 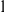 if 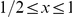.

In a traditional PGG experiment, some subjects form a group. Each subject is endowed with a certain amount of money, and they have to decide how much to invest in the public project, which is increased to a multiple of it and then split evenly among all subjects. So the gains of the subjects consist of two parts: the money left that they do not invest and the money gained from investing in the public project. For example, each of a four-member group is given 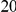 money units (MUs), and the money invested in the public project is doubled. If all members invest 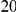 MUs, everyone will have 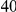 MUs. However, every invested MU only returns a half, and thus all members have an incentive to keep all money in pocket. If you defect by investing zero while every other member invests 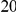 MUs, you will have 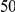 MUs while other members 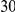 MUs per person. If all members defect, everyone ends up with 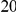 MUs and the benefit of the public project is forgone. Consequently a dilemma arises. Since every invested MU returns a half, from now on we call it a *linear* PGG, instead (Fig. 1).

In the linear PGG, investing nothing is the only equilibrium. That is, no one can gain more by investing more than zero no matter how much others invest. However, whether in linear PGG experiments or in real life, people often invest more than zero [9]. To better understanding people's behaviors, the *threshold* PGG is extensively researched (Fig. 1). In the threshold PGG, there exits a provision point or threshold value. If the total sum of the contributions is less than it, all contributions are lost, whereas if the total sum exceeds it, a fixed amount of the public good is gained. In contrast to the linear PGG, the threshold PGG has other equilibria except investing nothing. That is, any combination of contributions that sum to the provision point is an equilibrium. For example, each of a four-member group is given 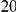 MUs, and when the money invested in the public project reached 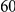 MUs every member is given extra 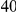 MUs. Then every member invests 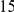 MUs is an equilibrium. Three investing 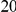 MUs and one investing zero is another equilibrium. A threshold PGG is a dilemma with a coordination game embedded in it [8].

However, most of social dilemmas in the real world are not with an obvious or clearly defined provision point. For example, in order to establish and maintain a public local library, those initial donations are important. Once the library starts to run, extra donations are also important for keep it running smoothly. But they are not as important as those that finally make possible the establishment of the library. Therefore, a tilted S-shaped continuous function such as a sigmoid function may provide a better model of many social dilemmas [8], [10]–[12]. We refer to a PGG with this kind of structure as a *sigmoid* PGG (Fig. 1). As pointed out in [11], the linear or threshold PGG is a simplification, or rather an extreme version of the sigmoid PGG.

So far, there have been very few efforts made to directly explore the effect of nonlinearity of the structures of public goods on the evolution of cooperation. In [10], a rather simple model was employed to independently analyze the accelerating, linear, and decelerating portions of the S-shaped function, so that the complexity of directly dealing with the S-shaped function itself was circumvented. In [12], the authors concluded by adopting replicator dynamics that the threshold PGG (therein is called the Volunteer's Dilemma) is a good approximation of any public goods games in which the public good is a nonlinear function of the number of cooperators (see further comparison to our analysis in section Results and Discussion). Here we will apply Darwinian dynamics [4], [13]–[16] to analyze the evolutionarily stable strategies (ESS) of these three kinds of PGGs, and try to show that the sigmoid PGG is really a more proper model for the evolution of cooperation within the well-mixed population, compared with the linear or threshold PGG in that it can reinforce our understanding of people's behaviors in the real world.

### Analysis

The pioneering definition of ESS, which is originated by Maynard Smith and Price, refers to a strategy that, when common, can resist the invasion of a minority of any other strategy [17]. Resistance to invasion is a static concept, since it says nothing about what would happen if the population starts at (or is perturbed to) a nearby point [15]. Therefore, an ESS which does not require convergence stability may be unattainable through strategy dynamics by natural selection. This leads to the proliferation of related terminology such as evolutionarily unbeatable strategy, 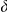-stability, internal stability, and evolutionarily singular strategy [18].

In contrast, Darwinian dynamics use a fitness-generating function (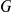-function) approach to continuous-trait evolutionary games [13], [14]. The 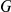-function allows for simultaneous consideration of population dynamics and strategy dynamics. An ESS is redefined as a coalition of strategies that is both convergent-stable and resistant to invasion, which is a natural extension of the original definition of Maynard Smith and Price. Those strategies consisting of an ESS are evolutionarily stable maxima on the adaptive landscape [4]. Here we adopt this definition of ESS.

In the following, we first introduce Darwinian dynamics and the extended concept of ESS. Then we analyze these three kinds of PGGs in this context. After the relatively simple linear and sigmoid PGGs are analyzed, the threshold PGG, which is not continuously differentiable so that the 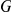-function approach cannot be directly applied to, is approximated by analyzing a class of PGGs with the structure of power functions.

### The 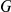-function Approach

*The* 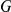 *-function approach* is mainly developed by Vincent, Brown, and their coauthors [4], [13], [14], [16]. We begin with introducing the fitness-generating function (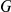-function). Assume that there are 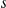 populations, and that the 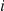-th population adopts the strategy 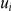 and its frequency is 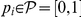. All strategies 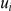
's are limited in the evolutionarily feasible set 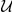. We set 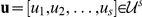 and 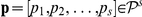. The 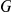-function 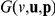 represents the fitness of the 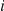-th population when the virtual variable 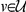 is replaced with 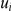.

Darwinian dynamics consist of population dynamics and strategy dynamics. In terms of the 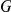-function 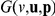, the population dynamics are given by
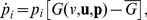
(1)where
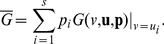
(2)When strategies 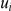
's do not evolve with time, they are equivalent to the replicator dynamics [19], [20]. The strategy dynamics are given by
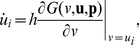
(3)where 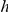 is a positive factor that influences the speed of the evolution of strategies [16]. In the special case that one extant strategy is invaded by one rare mutant strategy, they reduce to the adaptive dynamics [14], [18], [21], [22].

A non-trivial equilibrium point 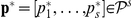 (reorder the indexes if necessary) is called an *ecologically stable equilibrium* point, if it satisfies that
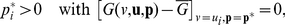
(4a)

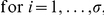


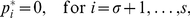
(4b)and that every trajectory starting from a point which is in 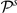 and near 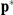 remains in 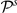 for all time and converges to 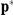 as time approaches infinity. The strategies corresponding to 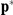 is denoted by 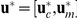, where
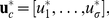
(5a)

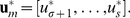
(5b)The coalition of strategies 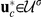 is defined as an *evolutionarily stable strategy* (ESS), if 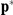 is an ecologically stable equilibrium point for any 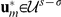. The *adaptive landscape* is simply a plot of 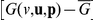 versus the virtual variable 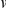 with 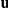 and 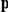 fixed. The *ESS Maximum Principle* [13] states that


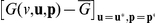
 *must take on its maximum value,* 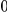 *, as a function of* 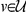 *at* 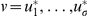.

Here we assume that the evolution of strategy is slower than that of population (but in all of the following invasion simulations we do not make this assumption), and focus on the ESS coalition of one strategy where 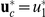 and 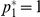. On the adaptive landscape, a stable minimum indicates an evolutionary branching point. The population which evolves to branching points may diverge into two separate populations or species with distinct strategies [18], [22]. Both unstable maxima and unstable minima are repelling points, and they should not be observed in nature [15]. An ESS is an global fitness maximum and convergently stable [14].

In the interior of 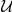, a necessary condition for 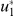 to resist the invasion of rare mutant strategies is given by
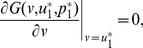
(6a)

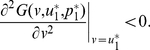
(6b)A necessary condition for the convergence stability of 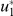 is given by
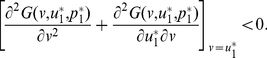
(7)


The linear PGG is played in a group of 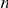 interacting members. Each member is endowed with 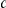 units of utility, and they have to decide how much to invest in the public project. The total units of utility invested in the public project is multiplied by a positive number 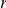 and then split evenly among all members. If 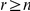, no member will lose anything no matter how much he invests. If 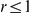, no member can gain more no matter how much he invests. So the number 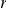 is restricted between one and 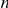. Group members benefit most when all cooperate, but each has an incentive to contribute nothing because every invested unit of utility only returns 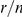 units of utility and thus cooperation incurs cost 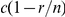 to himself. So the group will no doubt end up all members contributing nothing when they get experienced and the benefit of the public project is forgone. This is the dilemma all group members face. The interests of individuals totally contradict the interest of the group.

From now on we set 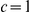 with no loss of generality, since it has no effect on the nature of the dilemma. We subsequently apply this 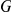-function approach to the aforementioned three kinds of PGGs, so as to analyze the dependence of cooperation levels on the structures of Public Goods.

For the PGG, if the populations are evolutionarily stable in the evolutionarily feasible set 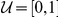, the expected contribution from any random group member is 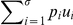. In a group of 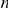 members, if the focal member decides to contribute 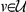, then the average contribution 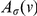 is given by
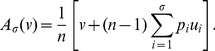
(8)Thus the return from the public good for the focal member is 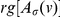, and the 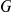-function is given by
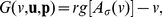
(9)where the function 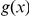 is supposed to represent the structure of the public good (Fig. 1).

### The Linear PGG

In the special case of the linear PGG of our interest here (Fig. 1), we set
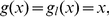
(10)and thus the 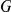-function is
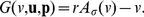
(11)It follows that
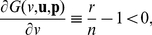
(12)which is independent of the composition of the population. Group members can always benefit more by reducing their contributions, so there exists no ESS in the interior of 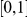.

**Figure 2:**
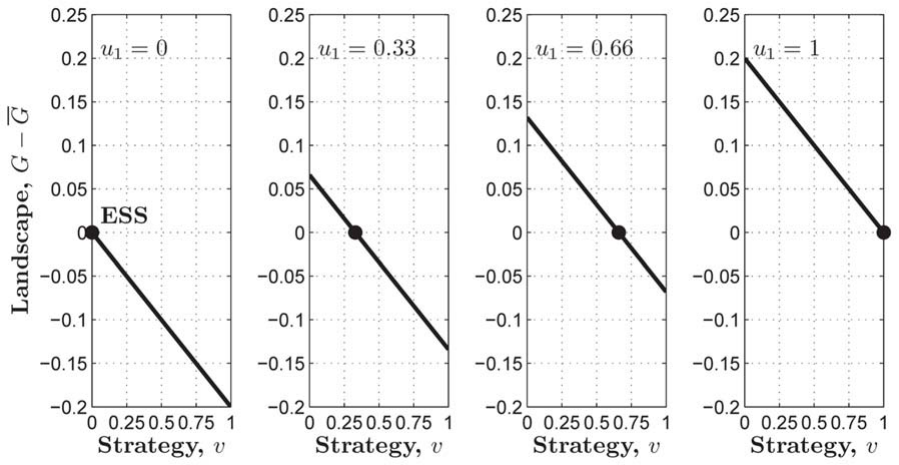
The adaptive landscapes in the linear PGG. 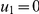
 is an ESS which sits at the top of the adaptive landscape. Parameters: 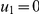, 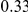, 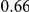, and 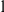; 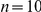; and 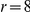.

However, this also gives us a hint that contributing nothing, where 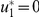 and 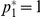, is the only possible ESS. Considering that the adaptive landscape
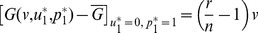
(13)reaches its global maximum, 0, in 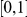 when 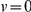 (Fig. 2), contributing nothing is surely an ESS for the linear PGG.

Similarly, we can conclude that another boundary value of 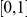, contributing all, where 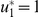 and 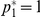, is not an ESS, since the adaptive landscape
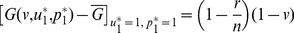
(14)reaches its global minimum, 0, in 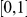 when 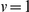 (Fig. 2).

**Figure 3:**
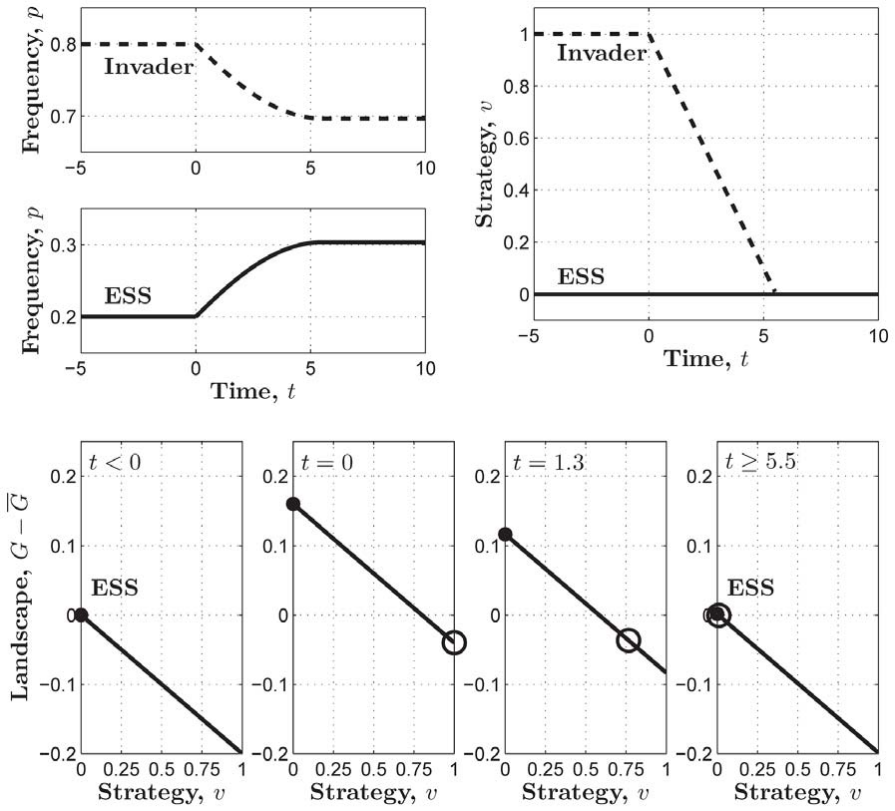
An invasion simulation of Darwinian dynamics of the linear PGG. (**Upper-left**) Evolution of the frequencies of the ESS and the invader strategy starting from 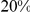 and 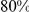 respectively. (**Upper-right**) Evolution of the ESS and the invader strategy starting from 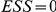 and 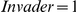 and ending up with the latter evolving to the former. (**Lower**) Evolution of the adaptive landscape and the two strategies: 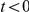 (i.e., before the invasion happens), 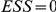 is the global maximum; 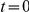 (i.e., the invasion happens), the adaptive landscape is elevated with 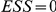 still being the global maximum and 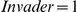 being the global minimum; 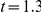, the invader strategy climbs up with the adaptive landscape going down; 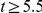, the invader strategy coincides with 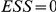 and reaches the top of the adaptive landscape, which falls back to the state before the invasion happens. Parameters: 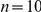, 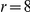, and 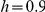.

A simulation of altruistic cooperators who contribute all (i.e., 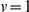) invading the population of defectors who contribute nothing (i.e., 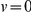) is shown in Fig. 3. The result shows that the ESS 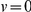 is rather robust against invasion. Yet this contradicts the fact that the mean contributions usually end up with between 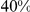 and 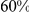 in experiments [9].

### The Sigmoid PGG

In the special case of the sigmoid PGG (Fig. 1), we set
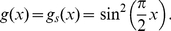
(15)Other functions with similar properties are of course possible, but not explored here for simplicity. Thereby the 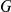-function is simplified as
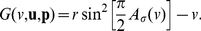
(16)


**Figure 4:**
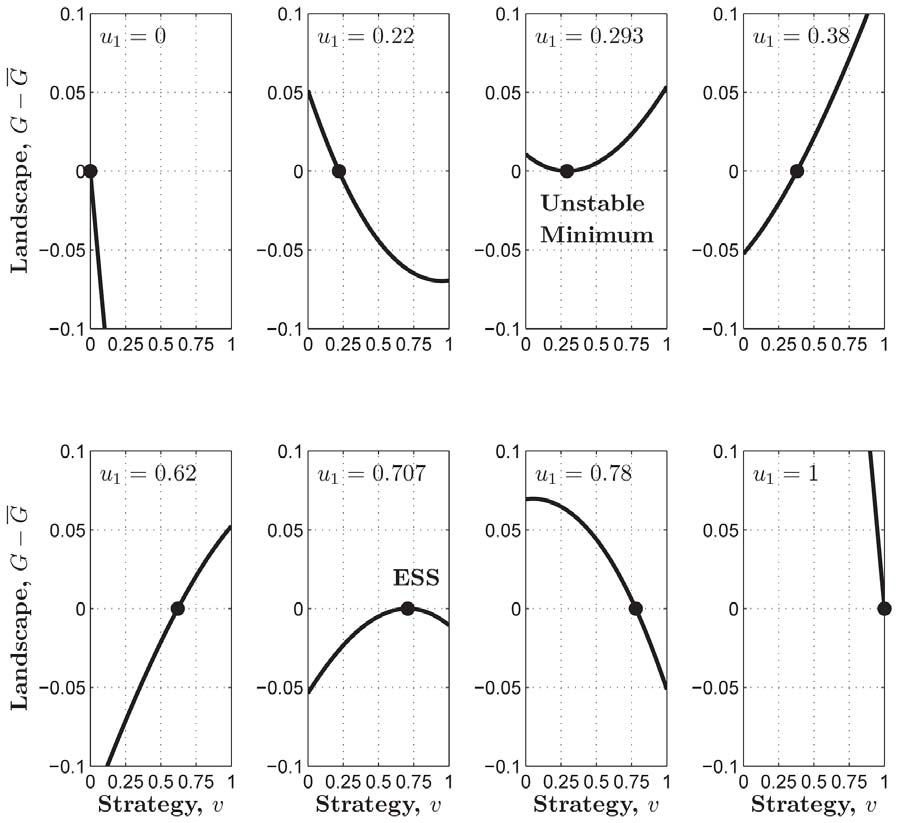
The adaptive landscapes in the sigmoid PGG. 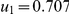
 is an ESS which sits at the top of the adaptive landscape. 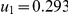 is an unstable minimum which sits at the bottom of the adaptive landscape. Parameters: 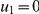, 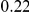, 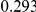, 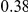, 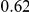, 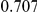, 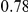, and 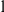; 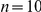; and 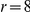.

We examine the one-strategy ESS (coalition of one strategy); that is, 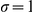. When 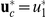 and 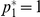, the 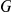-function (Fig. 4) is
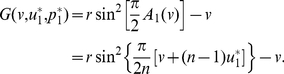
(17)


It follows that
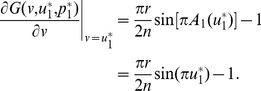
(18)If 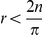, 
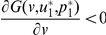
. We can verify that 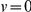 is the global maximum in 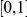 of the adaptive landscape
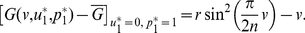
(19)Hence, if 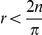, contributing nothing is also an ESS for the sigmoid PGG, just as in the case of the linear PGG.

When 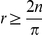, the equation 
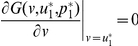
 has two solutions in 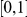:
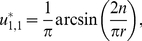
(20)and
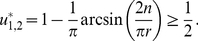
(21)We can identify 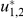 as an ESS candidate by verifying the following two conditions,
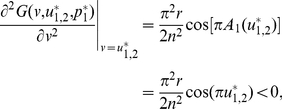
(22)and
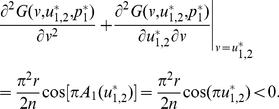
(23)Similarly, we can show that 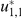 is an unstable fitness minimum. Hence there exists a stable state of the population contributing 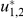, which is more than half, if the return rate 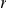 is relatively high.

**Figure 5:**
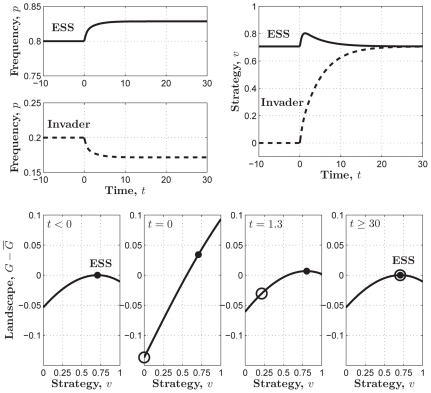
An invasion simulation of Darwinian dynamics in the sigmoid PGG. (**Upper-left**) Evolution of the frequencies of the ESS and the invader strategy starting from 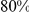 and 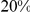 respectively. (**Upper-right**) Evolution of the ESS and the invader strategy starting from 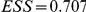 and 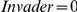 and ending up with the latter evolving to the former. (**Lower**) Evolution of the adaptive landscape and the two strategies: 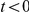 (i.e., before the invasion happens), 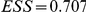 is the global maximum; 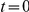 (i.e., the invasion happens), the adaptive landscape is reshaped with 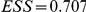 sitting at the left of the global maximum and 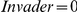 being the global minimum; 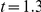, the two strategies climb up so that the adaptive landscape is reshaped with the global maximum sitting between the two strategies; 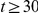, the two strategies coincide and reach the top, at 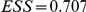, of the adaptive landscape, which falls back to the state before the invasion happens. Parameters: 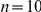, 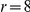, and 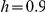.

A simulation of defectors who contribute nothing (i.e., 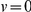) invading the population of individuals who play the ESS (i.e., 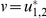) is shown in Fig. 5. The result shows that the ESS is surely able to resist the invasion.

### The Threshold PGG

**Figure 6:**
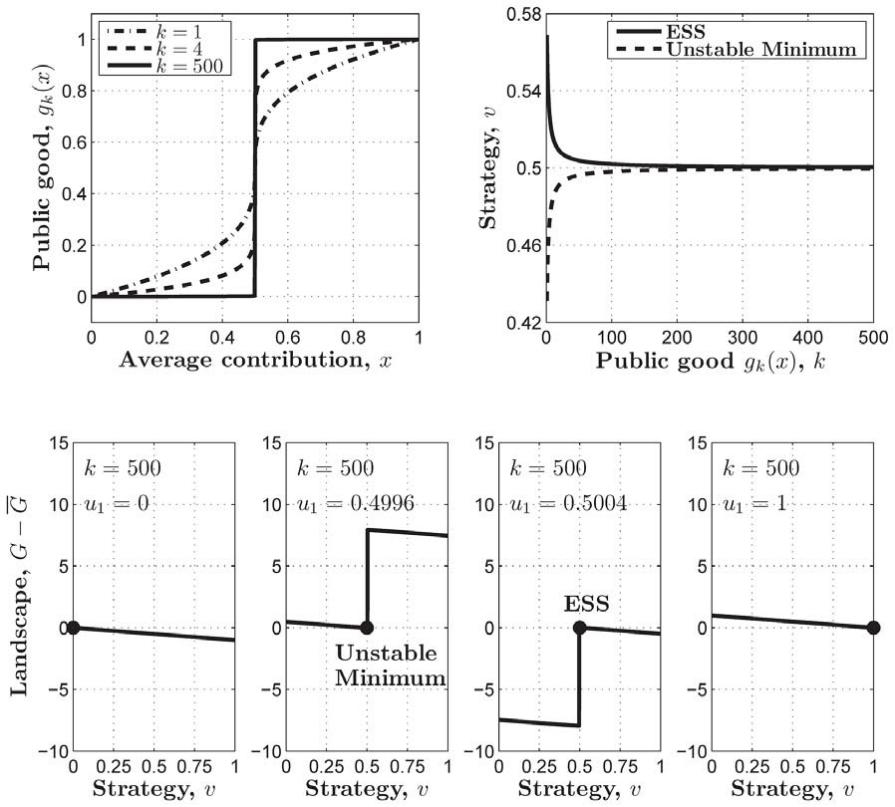
The approximate representative of the threshold PGG by a class of power functions. (**Upper-left**) 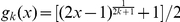 where 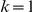, 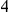, and 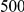. (**Upper-right**) the ESS and the unstable minimum as the functions of parameter 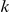. They are getting closer and closer with increasing 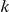. (**Lower**) the adaptive landscapes when 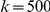, and 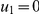, 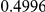, 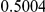, and 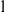. 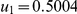 is an ESS which sits at the top of the adaptive landscape. 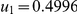 is an unstable minimum at the bottom of the adaptive landscape. Parameters: 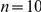, and 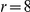.

For the special case of the threshold PGG (Fig. 1), we set
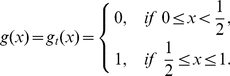
(24)Yet the discontinuity of 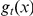 inhibits the application of Darwinian dynamics to our research into the process of evolution. Instead, we adopt a class of power functions 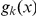, where 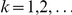, to approach function 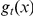 (Fig. 6); that is,
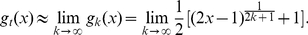
(25)Other functions with similar properties are of course possible, but not explored here for simplicity. Hence the 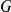-function can be expressed as
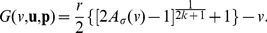
(26)


We still focus on the one-strategy ESS where 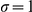. When 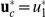 and 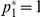, the adaptive landscape (Fig. 6) is
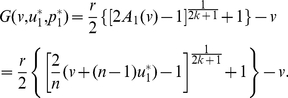
(27)


It follows that
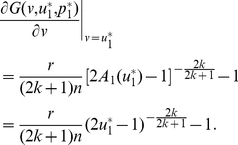
(28)The equation 
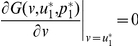
 also has two solutions in 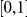:
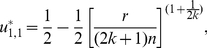
(29)and
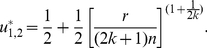
(30)


We can identify 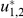 as an ESS candidate by verifying the following two conditions,
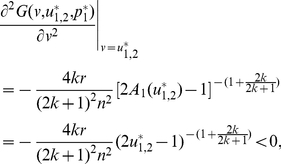
(31)and
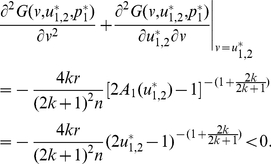
(32)Similarly, we can show that 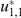 is an unstable fitness minimum. With increasing 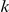, 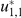 monotonically decreases, whereas 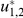 monotonically increases, and both approach the threshold value 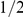 (Fig. 6).

**Figure 7:**
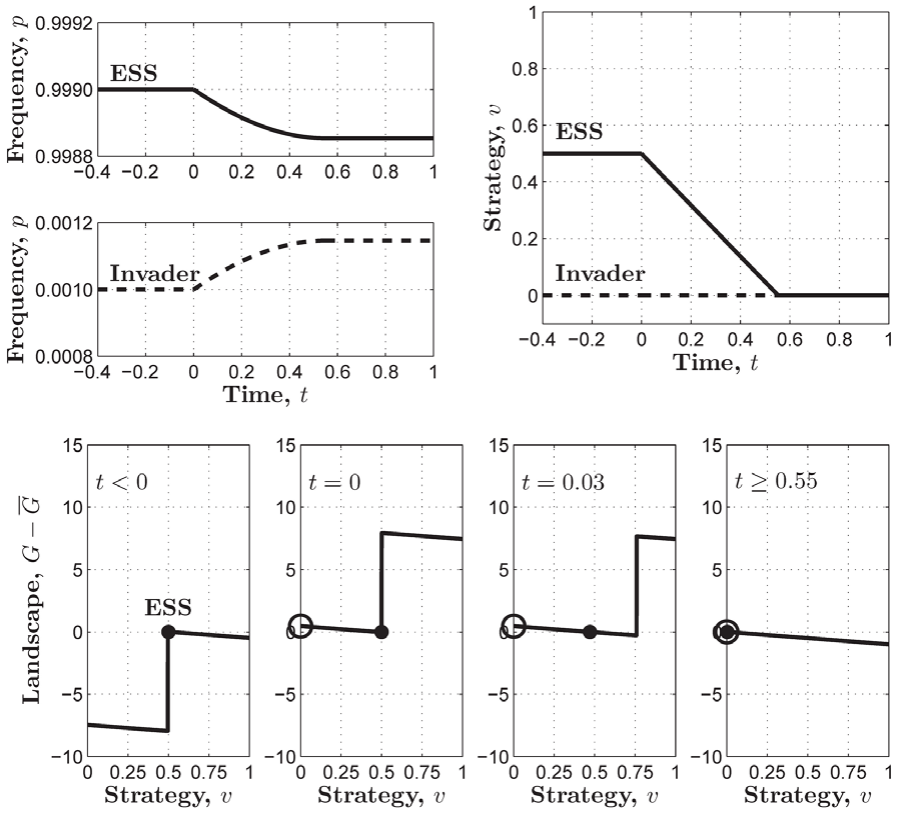
An invasion simulation of Darwinian dynamics in the threshold PGG which is approximated by 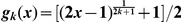 where 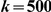. (**Upper-left**) Evolution of the frequencies of the ESS and the invader strategy starting from 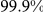 and 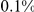 respectively. (**Upper-right**) Evolution of the ESS and the invader strategy starting from 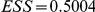 and 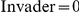 and ending up with the former evolving to the latter. (**Lower**) Evolution of the adaptive landscape and the two strategies: 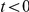 (i.e., before the invasion happens), 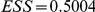 is the global maximum; 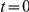 (i.e., the invasion happens), the adaptive landscape is elevated with 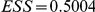 being the global minimum and 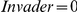 being the local maximum; 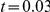, the “ESS” climbs up towards the invader strategy with the latter keeping sitting at the local maximum of the reshaped adaptive landscape; 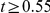, the “ESS” coincides with 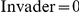 and reaches the top of the reshaped adaptive landscape, which means the success of the invader strategy and the failure of the “ESS”. Parameters: 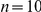, 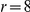, and 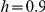.

Fig. 6 also shows that, in contrast to the ESS in the sigmoid PGG, here just on the left side of 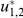 there exists a global minimum, which makes the ESS is rather fragile. This point is fully exposed in Fig. 7, where only 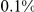 invaders of defectors who contribute nothing (i.e., 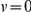) drove the whole population to the stable state of contributing nothing with a much faster speed relative to that in Fig. 3 or Fig. 5. Hence the threshold PGG basically does not have much advantage over the linear PGG.

## Results and Discussion

In summary, by adopting Darwinian dynamics, we have explored the significant effect of nonlinearity of the structures of public goods on the evolution of cooperation within the well-mixed population. The threshold PGG does not have much advantage over the linear PGG, whereas in the sigmoid PGG there exists a one-strategy ESS of the whole population contributing more than half. This suggests that the sigmoid PGG might be a more proper mathematical model for the research of the evolution of cooperation within the well-mixed population, and thereby may release researchers from the shackles of the linear or threshold PGG.

In contrast to most work in which replicator dynamics or adaptive dynamics were applied to the evolution of cooperation in social dilemmas [12], [22], here we adopt Darwinian dynamics mainly developed by Vincent, Brown, and their coauthors, which simultaneously consider the evolution of populations and strategies on a continuous adaptive landscape [4], [13], [14], [16]. In Darwinian dynamics, the concept of ESS is extended as a coalition of strategies that is both convergent-stable and resistant to invasion, whereas the original definition of ESS by Maynard Smith and Price might be unattainable through strategy dynamics by natural selection. This well-developed framework provides us with another wonderful mathematical tool for the research related to natural selection.

To our knowledge the only systematic theoretical analysis until now of the effect of nonlinearity of the structures of public goods on the evolution of cooperation is [12], in which a series of functions 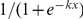 were adopted to explore the sigmoid PGG and their limit function when 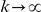 was used to approach the threshold PGG, and the authors concluded that the threshold PGG is a good approximation of any public goods games in which the public good is a nonlinear function of the number of cooperators. However, compared to Eqn. (28) we adopt here, 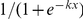 is not a good approximation due to its asymptotic nature. For example, this series of functions cannot represent full cooperation (or full defection) even though all individuals are cooperators (or defectors).

Both in the sigmoid PGG approximated by 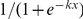 and in the threshold PGG approximated either by 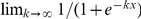 or by Eqn. (25), the ESS (note the different definition of ESS in our analysis from [12]) is accompanied by an unstable cooperation level (Figs. 6 and 7), which makes the ESS is rather fragile. In contrast, in the sigmoid PGG approximated here by Eqn. (15) the ESS is the only global extreme point in the interior of the evolutionarily feasible set 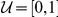 (Figs. 4 and 5). This suggests that the sigmoid PGG might be a more proper model for the evolution of cooperation within the well-mixed population, in that it hosts a non-trivial evolutionarily stable cooperation level when the return rate is relatively high, whereas the linear or threshold PGG never does.

Note that our results are reached within the well-mixed population. There exist different possibilities if we adopt other assumptions on the population, the group size, or the structure of the PGG. For example, within structured populations with different group sizes, the coexistence of cooperation and defection is possible even for the linear PGG due to noise underlying strategy adoptions [23]. The exploration of the linear PGG that requires a minimum collective investment to ensure any benefit shows that decisions within small groups under high risk significantly raise the chances of coordinating actions [24]. In addition, the relative size of the threshold value of the threshold PGG might also affect the evolution of cooperation within the structured population [25].

However, our work does show the significant effect of nonlinearity of the structures of public goods on the evolution of cooperation within the well-mixed population. Actually, when 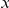 increases from 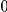 to 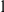, the slope of the S-shaped function 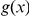 goes through a process from accelerating to decelerating. Simulations show that this property of 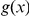 plays a key role for the existence of a robust ESS in the PGG within a well-mixed population. Naturally, an interesting future work might be to search for the optimal structure of public goods in the sense that complete cooperation is a robust global ESS in the PGG with this kind of structure, and the way to implement it in the real world.
